# Lifestyle Profiles and Their Sociodemographic Correlate in an Academic Community Sample

**DOI:** 10.3390/ijerph20010231

**Published:** 2022-12-23

**Authors:** Roberta Adorni, Elena Lonati, Francesco Zanatta, Michael Belingheri, Massimiliano Rossetti, Matteo Colleoni, Michele Augusto Riva, Paola Palestini, Patrizia Steca

**Affiliations:** 1 Department of Psychology, University of Milano-Bicocca, Piazza dell’Ateneo Nuovo, 20126 Milan, Italy; 2 School of Medicine and Surgery, University of Milano-Bicocca, Via Cadore, 48, 20900 Monza, Italy; 3 BASE Bicocca Sustainability Committee, University of Milano-Bicocca, Piazza dell’Ateneo Nuovo, 20126 Milan, Italy; 4 Department of Sociology and Social Research, University of Milano-Bicocca, Piazza dell’Ateneo Nuovo, 20126 Milan, Italy

**Keywords:** lifestyle, diet, physical activity, smoking, alcohol consumption, gender, age

## Abstract

Promoting healthy behaviors throughout life is an essential prevention tool. Prior research showed that unhealthy behaviors tend to co-occur and interplay. However, which behaviors co-occur most frequently and which sociodemographic variables are associated with specific clusters of unhealthy behavior are still being determined. This study aimed to identify different lifestyle profiles and analyze their associations with sociodemographic factors in an Italian academic community to plan targeted initiatives to promote healthy lifestyles. A sample of 8715 adults from an Italian university (mean age = 26 years; range = 18–76; 30% male) participated in an online survey in 2019. Four health-related behaviors were evaluated: diet, physical activity, smoking, and alcohol consumption. Lifestyle profiles were identified through cluster analysis. Then, a multinomial logistic regression was performed to explore the association among lifestyle profiles, sociodemographic variables (age, gender, and academic role), and body mass index (BMI). Results showed that older age was associated with the probability of belonging to the profile characterized by smoke addiction and regular alcohol consumption but also with the healthiest diet. The younger the age, the greater the probability of belonging to the most physically active profile. Men were more likely than women to belong to the lifestyle profile with the most regular alcohol consumption and the highest physical activity. Lower BMI was associated with the most physically active profile. This study shed light on factors associated with different co-occurring health-related behaviors that should be considered in planning effective communication strategies and preventive health interventions within the academic community.

## 1. Introduction

The most significant way to reduce the risk of several diseases is to promote a healthy lifestyle throughout the life span [1,2]. Prior research showed that behaviors such as diet, physical activity, cigarette smoking, and alcohol consumption tend to cluster in the general population [3,4,5,6,7,8]. The co-occurrence of different healthy or unhealthy behaviors results in a lifestyle that can have a multiplying effect on health compared to individual behaviors [2]. Therefore, studying the interplay among healthy behaviors and identifying different lifestyle profiles play a central role in preventing various diseases.

It is not clear which behaviors tend to be associated most frequently [6]. For example, some studies show that smoking behaviors tend to be associated with alcohol consumption [5,7,9], while an unhealthy diet tends to be associated with a sedentary lifestyle [7,9]. Other studies show an association between an unhealthy diet and smoking [5].

There is also considerable uncertainty about which sociodemographic variables are associated with specific groups of unhealthy behaviors. For example, the systematic review by Noble et al. [7] showed that men belonged more frequently to risk groups than women. The systematic review by Meader et al. [5] partially confirmed this observation. The role of age is still being determined, as some studies have shown an association between young age and belonging to riskier groups, while others have reported the opposite pattern [5,7].

The heterogeneity of the results reported in the previous literature depends on methodological factors, for example, the analytical strategy adopted and how behaviors are measured [10] and, more importantly, the specific sociodemographic and cultural characteristics of the population under study [8]. Therefore, identifying which health-related behaviors co-occur in a target sociodemographic and cultural context is essential in designing effective health intervention programs to modify risk behaviors [6,7,8].

Our study fits into this framework and aimed to explore the lifestyles of students and employees of a large Italian academic community to plan initiatives to promote healthy lifestyles for this type of community. Community participation is a crucial element of effective health promotion. Universities have a unique opportunity to promote a healthy lifestyle by implementing targeted internal policies. Our study responds to the intent of the Okanagan Charter for health-promoting universities and colleges [11]. This charter aims to stimulate concrete actions within universities to promote health and share good practices across the international academic communities. As underlined within the charter, health promotion requires an approach beyond focusing on individual behavior toward various interventions in social and environmental contexts. Intervening in the academic community, where individuals of different age groups and backgrounds study and work, is a valuable tool to promote healthy behaviors in their other living environments.

In line with the objectives of the Okanagan Charter, our study aimed to describe the lifestyle profiles of a specific academic community, thus giving specific insights to plan targeted actions. Such actions can create a campus culture of well-being and improve the health of people who live, learn, and work on university campuses.

Given the uncertainty of the prior literature and the paucity of studies focused on academic communities, this study aimed to quantify healthy behaviors and identify lifestyle profiles that simultaneously took into account diet, physical activity, cigarette smoking, and alcohol consumption in a sample of adults from our academic community. Moreover, it aimed to analyze the association between the sociodemographic variables (i.e., age, gender, academic role), BMI, and lifestyle profiles.

We adopted a person-centered approach [12] to assess how different health-related behaviors can form different lifestyle profiles. This approach grouped individuals based on their similarities. Therefore, it allowed researchers to explore the functioning of individuals from a more integrated perspective than the traditional approaches centered on singular variables, which consider components of the individual taken in isolation.

## 2. Materials and Methods

### 2.1. Participants and Procedure

The data were collected between May and June 2019 via an online survey. Eligible participants included all students and employees of the University of Milano-Bicocca, a large university in northern Italy. About 36,000 people were invited to participate via institutional email. The online survey was created, piloted, and administered using the LimeSurvey platform. The first page of the survey detailed the study’s objective and asked participants to provide their digital informed consent, declaring to have read and accepted the privacy regulation. The participation was anonymous and voluntary, and participants could abandon the research without consequences.

A total of 8744 volunteers completed the survey (response rate: about 25%). Table 1 illustrates the sociodemographic characteristics of the sample included in the analyses.

The research was carried out following The Code of Ethics of the World Medical Association (Declaration of Helsinki) for experiments involving humans. It was designed to provide information to the public health authority about the lifestyles among healthcare workers and students, in order to implement workplace health promotion policies. Before proceeding, we produced a formal document of collaboration with the Director of the University of Milano-Bicocca, attesting the purpose of the study and the treatment of the data according to the privacy policy. Moreover, we requested a legal opinion from the General Director of the University, and her opinion was favorable. Finally, we presented and discussed the project with the Director and President of the University Committee of BASE (Bicocca Alimentazione Sostenibilità ed Economia), who gave a favorable opinion.

### 2.2. Lifestyle Measures

#### 2.2.1. Diet

Participants reported the frequency of consumption of different food types (bread, pasta, rice, cold cuts, white meats, red meats, dairy products, eggs, fish, fruit and vegetables, legumes, salty snacks, and sweets) through a 5-point Likert scale, where 1 meant “More than once a day” and 5 meant “Never.” In addition, the participants reported which fats (olive oil, other vegetable fats, or butter/lard) they used most frequently for cooking and seasoning foods. Finally, they reported if they paid attention to their salt consumption through a 3-point Likert scale, where 1 meant “I have never paid attention” and 3 meant “I have always paid attention.” The items were taken from a survey of the National Institute of Statistics [13]. Regardless of how different eating behaviors were measured, we classified each specific consumption as adequate (score = 1) or inadequate (score = 0), following international [14] and national [15] guidelines.

To build a diet adequacy index for use in subsequent analyses, we added up the scores obtained for specific foods, similar to the MedDietScore scale [16].

Finally, to briefly describe the participants’ behavior, we classified their diet as inadequate (score between 1 and 9), sufficiently adequate (score between 10 and 15), and fully adequate (score ≥ 16).

#### 2.2.2. Physical Activity

Participants reported the frequency of three different types of physical activity (light, moderate, and intense) through a 4-point Likert scale, where 1 meant “Never” and 4 meant “Five or more days a week.” The items were based on a survey of the National Institute of Health [17]. Following the guidelines [18], light physical activity referred to daily movements, such as walking or cycling. Moderate physical activity, helpful in obtaining health benefits, was defined as that capable of increasing the heart rate and determining a mild subjective feeling of shortness of breath and warming up, allowing to increase the metabolism by 3–6 times compared to the rest situation. Moderate-intensity physical activity can be achieved with all sports and a large part of daily activities. High intensity is obtained instead when sweating and shortness of breath occur. We classified each response by assigning a score from zero to four, taking into account both intensity (light, moderate, intense) and frequency (zero to more than five times a week), following international [19] and national [17,18] guidelines. Guidelines were based on Global recommendations on physical activity for the health of the World Health Organization (WHO) published in 2010. WHO recommends practicing at least 150 min of moderate aerobic physical activity per week, at least 75 min of vigorous aerobic physical activity, or an equivalent combination of moderate and vigorous activity.

To build a physical activity adequacy index for use in subsequent analyses, we added the scores obtained for the single responses, similar to what we did for the diet.

Finally, to briefly describe the participants’ behavior, we classified their physical activity as inadequate (score between 0 and 1), adequate (score between 2 and 5), and intensive (score ≥ 6).

#### 2.2.3. Cigarette Smoking

Participants were asked whether they smoked or not. If yes, smoking addiction was measured using the Fagerström Test for Nicotine Dependence (FTND) [20]. It contains six items that evaluate the quantity of cigarette consumption, the compulsion to use, and dependence. In scoring the Fagerström Test for Nicotine Dependence, yes/no items are scored from 0 to 1, and multiple-choice items are scored from 0 to 3. The items are summed to yield a total score of 0–10. The higher the total Fagerström score, the more intense the individual’s physical dependence on nicotine.

We reversed the scores to have all lifestyles coded so that a low score indicates a behavior non-adherent to guidelines and a high score indicates an adherent behavior. Thus, a score of 0 identifies participants with a strong smoking addiction; a score of 10 identifies participants who did not smoke. Hence, the variable smoking considered the smoking habit and the level of smoking addiction.

To briefly describe the participants’ behavior, we classified smokers as non-adherent (score < 10) and nonsmokers as adherent to guidelines (score = 10).

#### 2.2.4. Alcohol Consumption

Participants were asked if they were teetotalers or used to drinking alcohol. If so, to assess their usual alcohol consumption, they were asked to report how many glasses of alcoholic beverages they drank on average during the week through an 8-point Likert scale, where 1 meant “16 or more glasses per day” and 8 meant “Less than one glass per day.” Thus, a score of 1 identified the participants with a strong habit of drinking alcohol (16 or more glasses per day); a score of 8 identified participants who consumed alcohol occasionally (less than one glass per day). A score of 9 was assigned to participants who abstained or had not drunk alcohol in the past 12 months. The items were taken from a survey of the National Institute of Statistics [13].

To briefly describe the participants’ behavior, we classified participants who drank at least one glass daily as non-adherent (score < 8). Teetotalers, those who had not drunk alcohol for at least 12 months, and participants who drank less than one drink per day were classified as adhering to the guidelines (scores between 8 and 9). This classification is coherent with national guidelines [21].

### 2.3. Data Analysis

We performed analyses with SPSS, version 26, Jamovi, version 1.6, and ROPstat [22], a statistical package for typological analyses.

We performed cluster analyses on the continuous scores of the four lifestyle variables, following the recommendations of Bergman [23]. Firstly, we z-standardized all four variables. Moreover, according to standard options [24], we performed a residue analysis (average squared Euclidean distance—ASED—less than 0.5). We identified 29 multivariate outliers (0.3% of the sample) and excluded them from the subsequent analyses. The outliers did not differ significantly from the 8715 included cases in age (U = 144,547.500; z = 1.402; *p* = 0.161), academic role (χ^2^(1,8744) = 0.567; *p* = 0.451), or BMI (U = 120,616.500; z = −0.271; *p* = 0.786), but they differed in gender (χ^2^(1,8744) = 13.883; *p* < 0.001). The percentage of males was higher among the excluded cases (62.1%) than in the included cases (30.3%).

We applied a two-step clustering procedure, combining Ward’s hierarchical and non-hierarchical k-means methods. We chose various solutions based on the size of the change in the explained error sum of squares percentage (%EESS) value between adjacent cluster solutions in the hierarchical method. Then, we used each solution as the initial cluster center for a non-hierarchical k-means clustering procedure. After the non-hierarchical clustering method, we used five indices to evaluate the optimal number of clusters to extract: a %EESS of at least 50, the point-biserial correlation coefficient, the modified Xie-Beni index of clustering adequacy, the Silhouette Coefficient, and the weighted mean of cluster homogeneity. The minimum value of the modified Xie-Beni index and the maximum of the other fit indices suggested the optimal number of clusters to retain, hence the best cluster solution. Another criterion for cluster solution retention was a reasonable cluster size (i.e., every cluster contained at least 5% of all the cases) [25].

We performed a multinomial logistic regression analysis. The variable “lifestyle profile” was the dependent variable (5 levels, one for each cluster identified by the typological analysis), and the sociodemographic variables were the predictors. Gender (male vs. female) and academic role (student vs. worker) were categorical predictors; age and BMI were covariates.

## 3. Results

### 3.1. Description of Lifestyles

Overall, most of the sample had a sufficiently healthy diet, did not smoke, and adhered to guidelines concerning alcohol consumption. The highest non-adherence percentage was found in physical activity, while the lowest non-adherence percentage was found in smoking. Despite the relatively low percentage of individuals not adhering to a healthful diet, only a tiny percentage of the sample had a diet that fully adhered to the guidelines (Table 2).

### 3.2. Identification of Lifestyle Profiles

**Figure 1:**
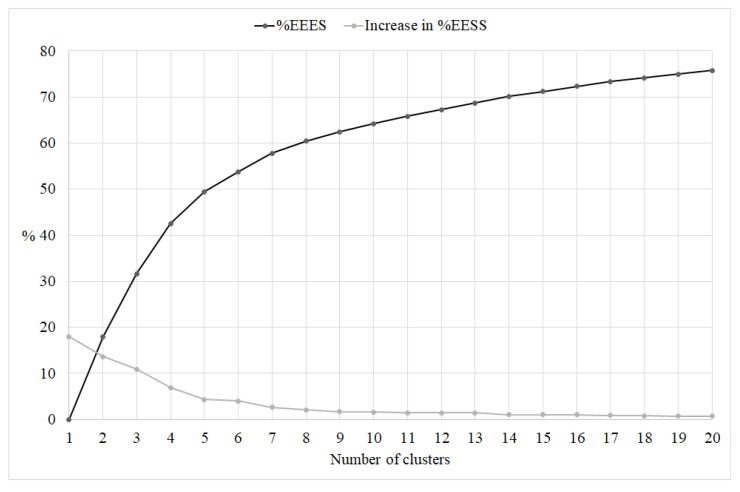
Scree-type plot for the cluster solutions. %EESS = explained error sum of squares percentage. Increase in %EESS = size of the change in the %EESS of adjacent solutions. The *x*-axis reports the number of clusters of each solution; the *y*-axis reports the percentages.

After evaluating the scree-type plot (Figure 1) showing the change in the %EESS by cluster solutions and based on the size of the change in the %EESS values, we retained the solutions from five to six clusters for further analysis.

Table 3 presents the fit indices of the retained cluster solutions. Three of the five indices are appropriate in the five-cluster solution. This solution showed more reasonable cluster sizes. Therefore, we identified the five-cluster solution as the optimal one.

**Figure 2:**
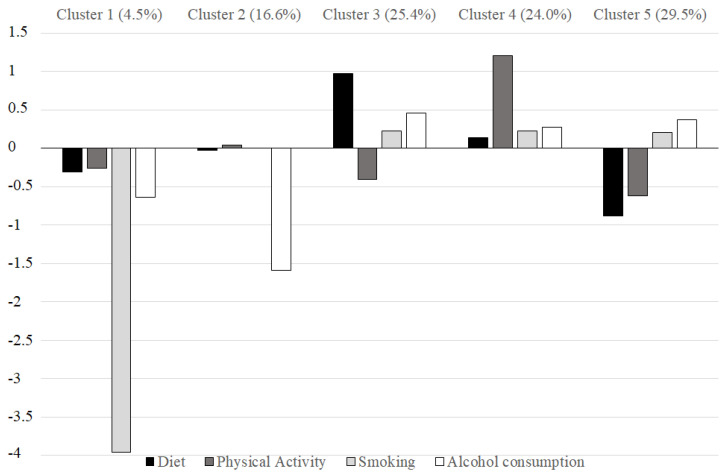
Z-scores of lifestyles for the final five-cluster solution.

Figure 2 presents the final cluster solution. The *y*-axis represents Z-scores. Because the clusters were defined using Z-scores for the total sample, each cluster’s mean z-scores indicate the distance between the cluster means and the total sample’s standardized mean. In other words, a Z-score between −0.5 and +0.5 denoted an average value (i.e., the “average participant” lifestyle). A Z-score over +0.5 denoted values above the sample mean (i.e., healthier than the average lifestyle).

Table 4 reports the lifestyle adequateness description for the identified clusters. Cluster 1 included individuals with medium behavior in diet and physical activity, medium-negative behavior in alcohol consumption, and markedly negative behavior in smoking. Nobody adhered to international guidelines regarding smoking. Cluster 2 included individuals with medium behavior in all lifestyles except alcohol consumption. Nobody adhered to international guidelines regarding alcohol consumption. Cluster 3 included participants with the best eating habits, a medium-positive smoking and alcohol consumption behavior, and a medium-negative behavior in physical activity. Cluster 4 included individuals with medium-positive behavior in all lifestyles and the highest adherence to international guidelines regarding physical activity. Cluster 5 included individuals with medium-positive behavior in smoking and alcohol consumption and negative diet and physical activity behavior. This cluster included the smallest percentage of participants adhering to a healthy diet and adequate physical activity.

### 3.3. Associations between Lifestyle Profile and Sociodemographic Indicators

**Figure 3:**
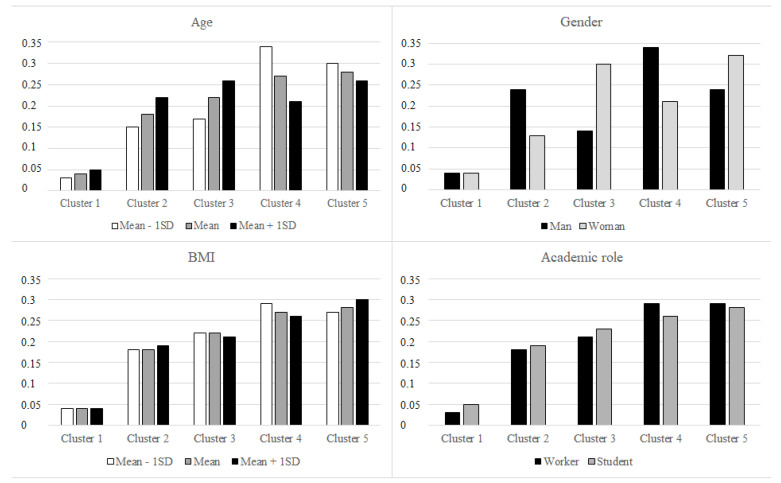
Distribution of the probability of belonging to each lifestyle profile as a function of sociodemographic variables and BMI.

The multinomial logistic regression model showed a good overall model fit based on the likelihood ratio test (χ^2^(16) = 706.998, *p* < 0.001) and the non-significance of the Pearson chi-square statistic (*p* > 0.05) and the Generalized Hosmer Lemeshow Test (*p* > 0.05). The model explained between 7.9% (Cox and Snell’s pseudo R^2^) and 8.4% (Nagelkerke’s pseudo R^2^) of the dependent variable variance. All independent variables made a unique, statistically significant contribution to the model (Table 5 and Figure 3).

An association emerged between age and the probability of belonging to a specific lifestyle profile. The older the age, the greater the probability of belonging to clusters 1, 2, and 3. The younger the age, the greater the probability of belonging to clusters 4 and 5. The ORs were close to 1, indicating tiny effect sizes.

The most robust predictor of lifestyle profile was gender. Men were more likely to belong to clusters 2 and 4 than women. Women were more likely to belong to clusters 3 and 5 than men.

Regarding the academic role, a significant difference emerged between cluster 1, more frequent among students, and cluster 4, more frequent among workers.

Regarding BMI, there was a significant difference between the probability of belonging to cluster 4 vs. clusters 1, 2, and 5. The lower the BMI, the greater the probability of belonging to cluster 4.

## 4. Discussion

This study described lifestyle in a sample from a large academic community and explored the associations between sociodemographic indicators and lifestyle profiles, adopting a person-centered approach.

Overall, most respondents exhibited behavior adhering to guidelines. In particular, 70.2% of the respondents had a sufficiently healthy diet, and 68.1% drank less than one drink per day. The university has a specific committee for “Water & Food,” which implemented a series of interventions aimed at promoting healthy and sustainable eating styles within the entire university population, acting on various levels, such as restaurants, bars, refreshment areas, and points reserved for the consumption of food brought from home. The interventions implemented include: installing water dispensers inside buildings, distributing steel water bottles at events, installing dining areas with microwave ovens and sinks for home food, and installing vending machines with healthy and sustainable products. The survey’s results indirectly suggest that these interventions stimulate healthy and sustainable food behaviors.

The highest adherence percentage (equal to 86.5%) was found in smoking behavior. The percentage of smokers (equal to 13.5%) was much lower than the national data (25.2%) [17]. This result may be explained by the presence in the university of internal policies to promote a healthy lifestyle, particularly the “tobacco-free policy.” Previous research showed that tobacco-free campus policies could reduce exposure to environmental tobacco smoke and prevent the onset of tobacco use. These policies can also promote smoking cessation and change social norms to de-normalize tobacco and increase the acceptability of smoking restrictions [26]. This evidence confirms that campus policies can help create a culture of well-being and improve people’s healthy behavior.

In line with national data [17] and international studies [27], the highest non-adherence percentage was found in physical activity. The university offers students and employees numerous sports activities, such as football, tennis, volleyball, basketball, gym, and bodyweight courses. The university also promotes events and tournaments and has a University Sports Center (CUS) that coordinates sports activities and events. University students and employees have discounted rates for participation in courses, events, and initiatives promoted and organized by the CUS. There are four sports facilities on the campus. Nevertheless, the results of this survey suggest that physical activity should be the preferred area for intervention shortly. With this in mind, starting from the 2021/2022 academic year, a Dual Career course was activated that is dedicated to university students with an athletic career. The university provides students who access the Dual Career program with a series of benefits and services to allow them to reconcile sporting commitments and university studies. This program aims to support the values of sports linked to well-being, healthy lifestyles, and correct behavior.

We identified five profiles, each characterized by its unique and specific lifestyle combination.

The configuration of the profiles confirmed the grouping of two behaviors suggested by the prior literature. The first grouping concerns smoking and alcohol consumption, which can be conceived as addictive behaviors requiring restraint or abstinence [3,7,9]. In the present study sample, these behaviors tended to cluster in adherence to international guidelines (clusters 3, 4, and 5) and non-adherence (cluster 1).

The second grouping concerns diet and physical activity, which can be conceived as behaviors that require an active commitment to promoting one’s health [3,7]. These behaviors tended to group in low adherence to international guidelines (clusters 1 and 5). However, a discrepancy emerged between diet adequacy and physical activity in clusters 3 and 4. Cluster 3 included the most significant number of individuals with a fully adequate diet and a “mean” adequate physical activity. Cluster 4 included the most significant number of individuals who performed intensive physical activity and had a “mean” adequate diet. This discrepancy could be because individuals who perform intensive physical activity usually consume more meats/protein foods than the others, thus having a diet that partially deviates from guidelines. Indeed, cluster 3 seemed to follow a healthy lifestyle with particular adherence to a healthy diet. In this group, physical activity could be part of behaviors aimed at psychophysical wellness without the need for nutrition that diverges from the guidelines for the general population. On the contrary, the carbohydrate + protein combination intake is a traditional strategy applied by endurance and strength-power athletes to ameliorate their performance. This approach increases muscle glycogen stores, minimizes muscle damage, and facilitates greater acute and chronic training adaptations [28,29]. However, pre- and post-exercise nutritional interventions (carbohydrate + protein or protein alone) need to be studied according to the International Society of Sports Nutrition (ISSN) guidelines and specific athlete characteristics.

Regarding the associations between the sociodemographic variables and lifestyle profiles, a minimal effect emerged between older age and the profiles characterized by smoking (cluster 1), highest alcohol consumption (cluster 2), and healthiest diet (cluster 3). The association between older age and smoking should not be surprising, although the prevalence of smokers among young people is usually higher than in older age groups. In this study, we considered smoking both in terms of a yes/no status and the level of addiction, which is usually higher in older than in young smokers [30]. The highest alcohol consumption in older people may be due to the moderate but regular consumption of alcohol (e.g., a glass of wine) during meals. Alcohol consumption in younger people is usually occasional because it is concentrated on the weekend [21]. Finally, the greatest attention to a healthy diet in older people may be related to a higher awareness and risk perception about the effects of unhealthy food on health and body shape.

Younger age was associated with a lifestyle characterized by the highest amount of physical activity (cluster 4) and a lifestyle characterized by the least healthy diet and the lowest amount of physical activity (cluster 5). Young people seemed divided into two groups: one who practiced intensive sport and took care of their lifestyle, and another with a sedentary lifestyle. The sedentary behavior of the second group could be due to uncorrected habits acquired in adolescence [31] or to the transition to university with an increase in the study burden or time spent in front of computers [32].

The strongest associations were found regarding gender. Men were much more likely than women to belong to the lifestyle profile with the highest alcohol consumption (cluster 2) and the highest physical activity (cluster 4). The first result confirmed a documented correlation between gender (male) and alcohol consumption in the general population [33] and students [34]. The second result was coherent with the prior literature highlighting gender disparity in interest and participation in physical activity. A recent review [35] showed that men tend to be more physically active than women throughout the life cycle. These differences may be related to different motivations for individuals to exercise. For men, the motivations tend to be intrinsic, such as improving health, preventing non-communicable diseases, and being competitive. For women, the motivations tend to be focused on social aspects. Interestingly, a recent study focused on university students [32] evidenced that the motive with the greatest difference between men and women was competition.

Our study also highlighted an association between academic role and belonging to the profile characterized by the highest physical activity. Workers were more likely to be in cluster 4 than students, who were more likely to be in cluster 1. This result is counterintuitive if we do not consider that it emerges net of the effect of age and gender and draws attention to a critical theme relating to the sedentary lifestyle of university students. A recent study mentioned above [32] underlined that university students represent a segment of the population that is most likely to adopt sedentary behaviors. It is worth pointing out that several studies suggest that moving to university makes students susceptible to adopting unhealthy routines, especially in terms of insufficient physical activity and an unhealthy diet [27,36,37]. Consistent with this evidence, cluster 1 of our study was characterized by less healthy behavior than cluster 4 in physical activity and other lifestyles.

Finally, the body mass index (BMI) parameter was analyzed in association with belonging to a cluster profile. Although with some limitations, this method is the most popular, shared, and advantageous anthropometric method for revealing the weight status distribution in a sample population. The results highlighted that participants with a lower BMI were more likely to belong to cluster 4. Cluster 4 consisted of people with medium-positive behavior in all lifestyles, including diet and physical activity. Indeed, a healthy diet and a physically active lifestyle are fundamental to weight control [38]. Moreover, people with higher BMI mainly belonged to cluster 5, where the slightest adherence to diet and physical activity was observed. Accordingly, it was demonstrated that people with excess weight often experience and internalize weight stigma, which significantly impacts their mental and physical health via adopting unhealthy eating behavior [39].

Our study has some limitations. First, information about lifestyles was self-reported and thus prone to information bias such as recall bias or social desirability bias. Ecologically valid methods might help evaluate the truthfulness of individuals’ reported information. Notwithstanding the methodological limitations of self-reported measures, they are suitable for providing essential steps in understanding a phenomenon [40]. They have substantial advantages, such as ease of use and an excellent cost-benefit ratio. Second, it cannot be excluded that the individuals most sensitive to the health issue and therefore attentive to their lifestyle participated in the survey. A final limitation of this study concerns the representativeness of the sample, which is very high in the case of employees and lower in the case of students, especially males.

Despite limitations, this study focused on a large sample of a specific community, providing relevant insights for future studies and interventions in universities. Moreover, exploring multiple co-occurrent health-related behaviors and using the person-centered analytical approach allowed us to investigate the individuals’ behavior from a more integrated perspective than the traditional approaches centered on singular variables, and they represent this study’s main strength. Information about whether and which lifestyles cluster together and which sociodemographic characteristics are associated with the riskiest clusters can facilitate identifying vulnerable population groups for targeting health promotion strategies and can contribute to developing effective and holistic preventive health interventions within the academic community.

## 5. Conclusions

Overall, our results show that individuals with specific sociodemographic characteristics within the academic community should be sensitized in different ways to the issue of healthy lifestyles. An approach based on the specific characteristics of a target group can be a promising strategy for promoting a healthy lifestyle. For example, older people should be sensitized to the health risks of smoking addiction, a sedentary lifestyle, and daily alcohol consumption. Young people should be sensitized to a healthy diet. More attention to a healthy diet and low alcohol consumption should be stimulated in men. In women and students, it would be helpful to stimulate greater awareness of the importance of regularly exercising. In this regard, universities could define their timetables in coordination with sports services. In addition, food education workshops could be organized to provide some milestones on buying healthy food, preparing healthy meals, and consuming seasonal fruit and vegetables. Future steps could also include implementing innovative technological tools (e.g., smartphone apps) that allow for the profiling of people and the consequent tailored communication of health claims. Prior research suggests the usefulness of mobile app-based health promotion strategies [41,42].

Community participation is a critical element of effective health promotion. Universities have a unique opportunity to promote a healthy lifestyle by implementing targeted internal policies. Intervening in the academic community, where individuals of different age groups and cultural backgrounds study and work, is a valuable tool for promoting healthy behaviors in their other living environments.

## Figures and Tables

**Table 1:** Sociodemographic characteristics of the sample (*n* = 8715).

Academic Role	Gender	*n* (%)	Response Rate (%)	Age Mean (SD) Range	BMI Mean (SD) Range
Students	Males	2101 (24.1)	15.9	23 (5.07) 18–68	23 (2.87) 15–35
	Females	5272 (60.5)	25.6	23 (4.89) 18–67	21 (3.06) 13–35
Administrative technical staff	Males	146 (1.7)	45.9	45 (8.74) 24–64	25 (2.88) 19–32
	Females	292 (3.4)	57.5	46 (8.92) 21–66	23 (3.56) 17–33
Ph.D. students, research fellows, postgraduates	Males	177 (2)	44.5	30 (5.55) 19–56	23 (2.87) 17–33
	Females	286 (3.3)	66.5	30 (5.45) 19–55	21 (2.76) 16–34
Researchers, professors	Males	213 (2.4)	38.0	49 (10.18) 28–76	25 (2.93) 19–34
	Females	228 (2.6)	55.9	50 (9.13) 27–75	22 (3.06) 16–32

**Table 2:** Descriptive statistics of the four lifestyle variables.

	Diet	Physical Activity	Smoking	Alcohol Consumption
Mean (SD)	11.18 (2.55)	2.24 (1.89)	9.68 (1.03)	8.02 (1.00)
Range	3–18	0–9	1–10	2.5–9
Skewness (SE)	−0.11 (0.03)	0.65 (0.03)	−4.11 (0.03)	−1.25 (0.03)
Kurtosis (SE)	−0.35 (0.05)	0.11 (0.05)	19.07 (0.05)	1.69 (0.05)
Behavior’s classification	NA = 25.7% Suff-A = 70.2% Fully A = 4.1%	NA = 36.4% A = 57.6% Intensive = 6.0%	NA = 13.5% A = 86.5%	NA = 31.9% A = 68.1%

Note. NA = non-adherent. Suff-A = sufficiently adherent. Fully A = fully adherent. A = adherent.

**Table 3:** Fit indices of the five- and six-cluster solution identified through K-means cluster analysis.

	5 Clusters	6 Clusters	Best Solution
%EESS	57.24	59.83	6 clusters
Point-biserial correlation coefficient	0.318	0.319	6 clusters
Modified Xie-Beni index	0.492	0.541	5 clusters
Silhouette Coefficient	0.575	0.572	5 clusters
Weighted mean of cluster homogeneity coefficients (HC, weights are cluster sizes)	0.856	0.804	5 clusters

**Table 4:** Sociodemographic description and adequateness of lifestyle for the clusters identified.

				% Healthy Lifestyles			
Cl	*n* (%)	Mean Age (SD)	*n* Male (%)	Diet	Physical Activity	Cigarette Smoking	Alcohol Consumption
1	393 (4.5%)	27.65 (10.28)	127 (32.3%)	227 ^a^ (57.8%) 12 ^b^ (3.1%)	188 ^c^ (47.8%) 10 ^d^ (2.5%)	0 (0%)	171 (43.5%)
2	1445 (16.6%)	27.67 (10.65)	649 (44.9%)	1083 (74.9%) 29 (2.0%)	1002 (69.3%) 46 (3.2%)	1108 (76.7%)	0 (0%)
3	2210 (25.4%)	27.41 (10.22)	377 (17.1%)	1960 (88.7%) 250 (11.3%)	1173 (53.1%) 0 (0%)	2079 (94.1%)	1948 (88.1%)
4	2094 (24.0%)	24.37 (7.26)	839 (40.1%)	1755 (83.8%) 64 (3.1%)	1628 (77.7%) 466 (22.3%)	1972 (94.2%)	1648 (78.7%)
5	2573 (29.5%)	25.13 (8.29)	645 (25.1%)	1094 (42.5%) 0 (0%)	1031 (40.1%) 0 (0%)	2380 (92.5%)	2167 (84.2%)

Note. This table classified each behavior according to whether participants met recommended guidelines. Percentages of adherent participants are reported. (^a^) = Participants sufficiently adherent to a healthy diet; (^b^) = participants fully adherent to a healthy diet. (^c^) = Participants who performed an adequate physical activity; (^d^) = participants who performed an intensive physical activity.

**Table 5:** Multinomial logistic regression results.

			95% CI						95% CI	
Predictor	Cluster Pairs	Log Odds Ratio	Lower	Upper	SE	Z	*p*	Odds Ratio	Lower	Upper
Age	1 vs. 4	0.06	0.04	0.07	0.01	6.62	<0 .001	1.06	1.04	1.07
	1 vs. 5	0.04	0.02	0.05	0.01	4.81	<0.001	1.04	1.02	1.05
	2 vs. 4	0.04	0.03	0.06	0.01	7.58	<0.001	1.05	1.03	1.06
	2 vs. 5	0.03	0.02	0.04	0.01	5.18	<0.001	1.03	1.02	1.04
	3 vs. 4	0.05	0.04	0.06	0.01	9.19	<0.001	1.05	1.04	1.06
	3 vs. 5	0.03	0.02	0.04	0.00	7.10	<0.001	1.03	1.02	1.04
	4 vs. 5	−0.02	−0.03	−0.01	0.01	−3.05	0.002	0.98	0.97	0.99
Gender	1 vs. 2	0.57	0.32	0.81	0.13	4.50	<0.001	1.76	1.38	2.26
	1 vs. 3	−0.80	−1.05	−0.55	0.13	−6.27	<0.001	0.45	0.35	0.58
	1 vs. 4	0.47	0.23	0.71	0.12	3.80	<0.001	1.59	1.25	2.03
	1 vs. 5	−0.30	−0.54	−0.06	0.12	−2.47	0.014	0.74	0.58	0.94
	2 vs. 3	−1.36	−1.52	−1.21	0.08	−16.91	<0.001	0.26	0.22	0.30
	2 vs. 5	−0.87	−1.01	−0.73	0.07	−11.86	<0.001	0.42	0.36	0.48
	3 vs. 4	1.26	1.11	1.41	0.08	16.70	<0.001	3.54	3.05	4.10
	3 vs. 5	0.49	0.35	0.64	0.08	6.55	<0.001	1.64	1.41	1.90
	4 vs. 5	−0.77	−0.90	−0.64	0.07	−11.52	<0.001	0.46	0.41	0.53
AR	1 vs. 4	0.51	0.08	0.94	0.22	2.31	0.021	1.66	1.08	2.56
BMI	1 vs. 4	0.05	0.01	0.08	0.02	2.55	0.011	1.05	1.01	1.09
	2 vs. 4	0.03	0.01	0.05	0.01	2.42	0.016	1.03	1.01	1.05
	4 vs. 5	−0.03	−0.05	−0.01	0.01	−3.11	0.002	0.97	0.95	0.99

Note. The table shows, for each predictor (“Predictor” column), all the statistically significant comparisons between the pairs of clusters (“Clusters” column, for example, 1 vs. 4 indicates that the difference between cluster 1 and cluster 4 was statistically significant). CI = confidence interval; SE = standard error; AR = academic role.

## Data Availability

The data presented in this study are available on request from the corresponding author. The data are not publicly available due to privacy and ethical restrictions.

## References

[1] Li Y., Pan A., Wang D.D., Liu X., Dhana K., Franco O.H., Kaptoge S., Di Angelantonio E., Stampfer M., Willett W.C. (2018). Impact of Healthy Lifestyle Factors on Life Expectancies in the US Population. Circulation.

[2] Loef M., Walach H. (2012). The combined effects of healthy lifestyle behaviors on all cause mortality: A systematic review and meta-analysis. Prev. Med..

[3] Adorni R., Zanatta F., D’Addario M., Atella F., Costantino E., Iaderosa C., Petarle G., Steca P. (2021). Health-Related Lifestyle Profiles in Healthy Adults: Associations with Sociodemographic Indicators, Dispositional Optimism, and Sense of Coherence. Nutrients.

[4] Mawditt C., Sacker A., Britton A., Kelly Y., Cable N. (2016). The clustering of health-related behaviours in a British population sample: Testing for cohort differences. Prev. Med..

[5] Meader N., King K., Moe-Byrne T., Wright K., Graham H., Petticrew M., Power C., White M., Sowden A.J. (2016). A systematic review on the clustering and co-occurrence of multiple risk behaviours. BMC Public Health.

[6] Morris L.J., D’Este C., Sargent-Cox K., Anstey K.J. (2016). Concurrent lifestyle risk factors: Clusters and determinants in an Australian sample. Prev. Med..

[7] Noble N., Paul C., Turon H., Oldmeadow C. (2015). Which modifiable health risk behaviours are related? A systematic review of the clustering of Smoking, Nutrition, Alcohol and Physical activity (‘SNAP’) health risk factors. Prev. Med..

[8] Rabel M., Laxy M., Thorand B., Peters A., Schwettmann L., Mess F. (2018). Clustering of Health-Related Behavior Patterns and Demographics. Results From the Population-Based KORA S4/F4 Cohort Study. Front. Public Health.

[9] De Vries H., van’t Riet J., Spigt M., Metsemakers J., van den Akker M., Vermunt J.K., Kremers S. (2008). Clusters of lifestyle behaviors: Results from the Dutch SMILE study. Prev. Med..

[10] McAloney K., Graham H., Law C., Platt L. (2013). A scoping review of statistical approaches to the analysis of multiple health-related behaviours. Prev. Med..

[11] Okanagan Charter: An International Charter for Health Promoting Universities & Colleges. https://open.library.ubc.ca/cIRcle/collections/53926/items/1.0132754.

[12] Magnusson D., Cairns R.B., Bergman L.R., Kagan J. (1998). The Logic and Implications of a Person-Oriented Approach. Methods and Models for Studying the Individual.

[13] Indagine Multiscopo Sulle Famiglie: Aspetti Della Vita Quotidiana—Parte Generale. https://www.istat.it/it/archivio/91926.

[14] World Health Organization (WHO) (2018). Healthy Diet: Key Facts. https://www.who.int/news-room/fact-sheets/detail/healthy-diet.

[15] Ministero Della Salute Linee Guida per Una Sana Alimentazione. https://www.salute.gov.it/portale/documentazione/p6_2_2_1.jsp?lingua=italiano&id=2915.

[16] Trichopoulou A., Costacou T., Bamia C., Trichopoulos D. (2003). Adherence to a Mediterranean Diet and Survival in a Greek Population. N. Engl. J. Med..

[17] EpiCentro Sorveglianza Passi. https://www.epicentro.iss.it/passi/.

[18] EpiCentro Attività fisica: Parametri e Livelli Consigliati e Ricadute Sullo Stato di Salute. https://www.epicentro.iss.it/attivita_fisica/livelli-consigliati.

[19] Physical Activity. http://www.who.int/news-room/fact-sheets/detail/physical-activity.

[20] Heatherton T.F., Kozlowski L.T., Frecker R.C., Fagerstrom K.-O. (1991). The Fagerström Test for Nicotine Dependence: A revision of the Fagerstrom Tolerance Questionnaire. Br. J. Addict..

[21] Il Consumo di Alcol in Italia. https://www.istat.it/it/archivio/198903.

[22] Vargha A., Torma B., Bergman L.R. (2015). ROPstat: A general statistical package useful for conducting person-oriented analysis. J. Pers. Res..

[23] Bergman L.R., Nurmi J.-E., Eye A.A. (2012). von I-States-as-Objects-Analysis (ISOA): Extensions of an Approach to Studying Short-Term Developmental Processes by Analyzing Typical Patterns. Int. J. Behav. Dev..

[24] Bergman L.R., Magnusson D., El Khouri B.M. (2003). Studying Individual Development in an Interindividual Context: A Person-Oriented Approach.

[25] Mahoney J.L., Stattin H., Magnusson D. (2001). Youth recreation centre participation and criminal offending: A 20-year longitudinal study of Swedish boys. Int. J. Behav. Dev..

[26] Braverman M.T., Ceraso M., Sporrer F., Rockler B.E. (2020). Five-year changes in support for tobacco control policy options among students, faculty and staff at a public university. Prev. Med..

[27] Aceijas C., Waldhäusl S., Lambert N., Cassar S., Bello-Corassa R. (2016). Determinants of health-related lifestyles among university students. Perspect. Public Health.

[28] Kerksick C.M., Arent S., Schoenfeld B.J., Stout J.R., Campbell B., Wilborn C.D., Taylor L., Kalman D., Smith-Ryan A.E., Kreider R.B. (2017). International society of sports nutrition position stand: Nutrient timing. J. Int. Soc. Sport. Nutr..

[29] Vitale K., Getzin A. (2019). Nutrition and Supplement Update for the Endurance Athlete: Review and Recommendations. Nutrients.

[30] Gallus S., Pacifici R., Colombo P., La Vecchia C., Garattini S., Apolone G., Zuccaro P. (2005). Tobacco dependence in the general population in Italy. Ann. Oncol..

[31] Martins J., Marques A., Gouveia R., Carvalho F., Sarmento H., Valeiro M.G. (2022). Participation in Physical Education Classes and Health-Related Behaviours among Adolescents from 67 Countries. Int. J. Environ. Res. Public Health.

[32] Carballo-Fazanes A., Rico-Díaz J., Barcala-Furelos R., Rey E., Rodríguez-Fernández J.E., Varela-Casal C., Abelairas-Gómez C. (2020). Physical Activity Habits and Determinants, Sedentary Behaviour and Lifestyle in University Students. Int. J. Environ. Res. Public Health.

[33] Erol A., Karpyak V.M. (2015). Sex and gender-related differences in alcohol use and its consequences: Contemporary knowledge and future research considerations. Drug Alcohol Depend..

[34] Belingheri M., Facchetti R., Scordo F., Butturini F., Turato M., De Vito G., Cesana G., Riva M.A. (2019). Risk behaviors among Italian healthcare students: A cross-sectional study for health promotion of future healthcare workers. Med. Lav..

[35] Rosenfeld C.S. (2016). Sex-dependent differences in voluntary physical activity. J. Neurosci. Res..

[36] Deforche B., Van Dyck D., Deliens T., De Bourdeaudhuij I. (2015). Changes in weight, physical activity, sedentary behaviour and dietary intake during the transition to higher education: A prospective study. Int. J. Behav. Nutr. Phys. Act..

[37] Deliens T., Deforche B., De Bourdeaudhuij I., Clarys P. (2015). Determinants of physical activity and sedentary behaviour in university students: A qualitative study using focus group discussions. BMC Public Health.

[38] Riebe D., Greene G.W., Ruggiero L., Stillwell K.M., Blissmer B., Nigg C.R., Caldwell M. (2003). Evaluation of a Healthy-Lifestyle Approach to Weight Management. Prev. Med..

[39] Brown A., Flint S.W., Batterham R.L. (2022). Pervasiveness, impact and implications of weight stigma. eClinicalMedicine.

[40] Paulhus D.L., Vazire S. (2007). The Self-Report Method. Handbook of Research Methods in Personality Psychology.

[41] Cotten E., Prapavessis H. (2016). Increasing Nonsedentary Behaviors in University Students Using Text Messages: Randomized Controlled Trial. JMIR Mhealth Uhealth.

[42] Lee M., Lee H., Kim Y., Kim J., Cho M., Jang J., Jang H. (2018). Mobile App-Based Health Promotion Programs: A Systematic Review of the Literature. Int. J. Environ. Res. Public Health.

